# Post-infarction cardiac remodeling—differential biomarkers for left atrial and left ventricular remodeling

**DOI:** 10.1007/s00109-021-02099-7

**Published:** 2021-06-04

**Authors:** Simina-Ramona Selejan, Mathias Hohl, Michael Böhm

**Affiliations:** Klinik für Innere Medizin III, Universitätsklinikum des Saarlandes und Medizinische Fakultät der Universität des Saarlandes (Saarland University Hospital and Saarland University Faculty of Medicine), Saar 66421 Homburg, Germany

Despite advances in myocardial infarction (MI) treatment, subsequent cardiac remodeling is still a relevant clinical problem. Post-infarction remodeling not only comprises left ventricular (LV) injury like scar formation, but also remodeling of the remote non-infarcted LV tissue and the left atrium (LA). The factors leading to atrial remodeling in MI are not well established. It is generally assumed that poor LV myocardial perfusion indirectly influences LA remodeling by causing LV dysfunction [1]. Still, relatively few pre-clinical and clinical studies take post-infarction atrial remodeling into consideration. Redondo et al. [2] took this additional step and investigated potential cardiac biomarkers for their usefulness in post-infarction remodeling for both left-sided heart chambers: Galectin-3 and sRAGE.

Galectin-3 is a lectin family adhesion molecule usually secreted from immune cells [3]. It is involved in various pathogenetic mechanisms promoting inflammation [4]. RAGE, the main receptor for advanced glycation end products (AGEs), is considered to not only be involved in diabetes-associated complications but a wide variety of diseases [5]. It is cleaved by metalloproteases to its soluble circulating form sRAGE, which has protective properties by binding and “neutralizing” AGEs [6]. Therefore, both Galectin-3 and sRAGE are circulating proteins easily measured in patient blood samples. Nonetheless, while Galectin-3 is regarded as a pro-remodeling factor, sRAGE is generally considered an anti-remodeling agent [4–6].

In this issue of the *Journal of Molecular Medicine*, Redondo et al. [2] describe baseline Galectin-3 levels as predictors of adverse post-MI LV remodeling, while sRAGE levels exhibit an inverse beneficial relationship with LA remodeling 6 months after MI. Galectin-3 being an independent predictive factor for LV remodeling is consistent with previous reports [7]. However, sRAGE plasma levels demonstrating an inverse relationship with LA remodeling is a novel observation. What are the potential mechanistic explanations for those results? Mechanistic contributors are often potential biomarkers. Although not obvious at first glance, both investigated biomarkers are receptors for AGEs. Galectin-3 facilitates binding of AGEs to their receptor RAGE [8], thereby promoting inflammation. In contrast, sRAGE prevents RAGE activation by binding AGEs and “neutralizing” them [5, 6], thus suppressing tissue remodeling. Surprising is the observation that each of the two proteins is, apart from their principally opposite mode of action, a biomarker for only one of the two left heart chambers post-infarction. Galectin-3 enhances macrophage and mast cell infiltration into the infarcted myocardium propagating inflammation-induced fibrosis [4], which is the probable mechanistic explanation. Both Galectin-3 and sRAGE however predict catheter ablation outcomes in atrial fibrillation without MI [9, 10], but only sRAGE is associated with atrial remodeling after MI [2]. AGE-sRAGE ratios being additionally directly related with LA enlargement, the assumption arises that AGEs could promote LA remodeling after MI and sRAGE could serve as decoy receptor counteracting AGEs [2]. Which leads to the question: Are AGEs differentially distributed in the heart after MI? Some evidence points to this. Despite the difficulty in obtaining human atrial tissue, it has been reported that AGEs levels are higher in the LA appendage of patients with atrial fibrillation than in controls [11] and that AGEs cause collagen cross-linking, which makes it difficult for collagenases to break down fibrosis [12]. At least one of the known AGEs, carboxymethyllysine (CML), accumulates in the atrium rather than in the ventricle after myocardial infarction [13], indicating that AGEs and sRAGE might play a greater role in atrial remodeling than in ventricular remodeling in this setting.

The present study adds another piece to the puzzle to the overall picture of MI-induced atrial and ventricular remodeling. Therefore, it might be worth investigating whether these two biomarkers might be actually indicating underlying mechanistic pathways. Sequential blood sampling would be important for the design of new studies, because it has already been shown that proteases such as MMP-9 are activated in acute MI and not only cleave RAGE and form sRAGE [6] but also degrade Galectin-3 [14], which somewhat diminishes the impact of early MI Galectin-3 and sRAGE levels with respect to their relevance for long-term remodeling.
